# Are *pvcrt-o* and *pvmdr1* Gene Mutations Associated with *Plasmodium vivax* Chloroquine-Resistant Parasites?

**DOI:** 10.3390/biomedicines12010141

**Published:** 2024-01-09

**Authors:** Rebecca de Abreu-Fernandes, Natália Ketrin Almeida-de-Oliveira, Aline Rosa de Lavigne Mello, Lucas Tavares de Queiroz, Jacqueline de Aguiar Barros, Bárbara de Oliveira Baptista, Joseli Oliveira-Ferreira, Rodrigo Medeiros de Souza, Lilian Rose Pratt-Riccio, Patrícia Brasil, Cláudio Tadeu Daniel-Ribeiro, Maria de Fátima Ferreira-da-Cruz

**Affiliations:** 1 Laboratório de Pesquisa em Malária, Instituto Oswaldo Cruz, Fundação Oswaldo Cruz (Fiocruz), Rio de Janeiro 21041-361, Brazil; rebeccasantos@aluno.fiocruz.br (R.d.A.-F.); nataliaketrin@gmail.com (N.K.A.-d.-O.); alinelavigne@uol.com.br (A.R.d.L.M.); lucasqueiroz@aluno.fiocruz.br (L.T.d.Q.); barros.jacqueline@gmail.com (J.d.A.B.); bbaptista22@gmail.com (B.d.O.B.); riccio@ioc.fiocruz.br (L.R.P.-R.);; 2 Centro de Pesquisa, Diagnóstico e Treinamento em Malária (CPD-Mal), Reference Laboratory for Malaria in the Extra-Amazonian Region for the Brazilian Ministry of Health, Secretaria de Vigilância Sanitária & Fiocruz, Rio de Janeiro 21041-361, Brazil; 3 Núcleo de Controle da Malária/Departamento de Vigilância Epidemiológica/Coordenação Geral de Vigilância em Saúde/SESAU-RR, Boa Vista 69305-080, Brazil; 4 Laboratório de Imunoparasitologia, IOC, Fiocruz, Rio de Janeiro 21041-361, Brazil; lila@ioc.fiocruz.br; 5 Laboratório de Doenças Infecciosas da Amazônia Ocidental, Universidade Federal do Acre, Campus Floresta, Cruzeiro do Sul 69980-000, Brazil; rodrigo.souza@ufac.br; 6 Instituto Nacional de Infectologia Evandro Chagas, Fiocruz, Rio de Janeiro 21040-361, Brazil

**Keywords:** chloroquine, chemoresistance, malaria, *P. vivax*, *pvcrt-o*, *pvmdr1*

## Abstract

(1) Background: Malaria remains a significant global public health issue. Since parasites quickly became resistant to most of the available antimalarial drugs, treatment effectiveness must be constantly monitored. In Brazil, up to 10% of cases of vivax malaria resistant to chloroquine (CQ) have been registered. Unlike *P. falciparum*, there are no definitive molecular markers for the chemoresistance of *P. vivax* to CQ. This work aimed to investigate whether polymorphisms in the *pvcrt-o* and *pvmdr1* genes could be used as markers for assessing its resistance to CQ. (2) Methods: A total of 130 samples from *P. vivax* malaria cases with no clinical and/or parasitological evidence of CQ resistance were studied through polymerase chain reaction for gene amplification followed by target DNA sequencing. (3) Results: In the *pvcrt-o* exons, the K10 insert was present in 14% of the isolates. Regarding *pvmdr1*, T958**M** and F1076**L** haplotypes showed frequencies of 95% and 3%, respectively, while the SNP Y976**F** was not detected. (4) Conclusions: Since K10-*pvcrt-o* and F1076**L**/T958**M**-*pvmdr1* polymorphisms were detected in samples from patients who responded well to CQ treatment, it can be concluded that mutations in these genes do not seem to have a potential for association with the phenotype of CQ resistance.

## 1. Introduction

Malaria is a major important public health problem worldwide. According to the World Malaria Report from the World Health Organization (WHO), there were 247 million new cases and 619 thousand malaria-related deaths in 2021 [1]. *Plasmodium vivax* is the most widely distributed species causing most malaria cases in Asia and South America [2,3]. Since 2007, Brazil has observed a stable incidence rate of malaria cases, with *P. vivax* accounting for about 80% of cases. This trend continued in 2022. The country recorded around 129,000 malaria cases, of which around 83% were attributable to *P. vivax* and 16%, to *P. falciparum* [4]. In the last 60 years, the combination of chloroquine (CQ) and primaquine (PQ) has been used in Latin America to radically cure *P. vivax*, i.e., eradicate the blood forms and hepatic hypnozoites [5]. However, emerging resistance to antimalarial drugs may threaten malaria control programs [1].

The first report of *P. vivax* resistance to chloroquine (CQR) in Papua New Guinea dates from 1989 [6], 30 years after resistance reports for *P. falciparum* [7,8]. In Brazil, the first cases of *P. vivax* resistant to CQ were described in Manaus, Amazonas, in 1999 [9]. This timeline reflects the emergence of chloroquine resistance in *P. vivax* occurring later than in *P. falciparum.* Since then, studies have reported a 10% prevalence of CQR [10] and a recurrence or reemergence of *P. vivax* in 5.2% of cases in the same region [11], threatening current international efforts to control and eliminate malaria [1]. Given the emerging risk of drug resistance, drug efficacy monitoring studies using molecular markers represent an important tool for refining CQR surveillance and validating potential molecular markers associated with the *P. vivax* resistance phenotype through mutated single nucleotide polymorphisms (SNPs) in related genes [12].

Currently, the mechanisms of resistance of *P. vivax* to antimalarial drugs are still unclear due to the lack of continuous in vitro culture systems and the possible involvement of multigenic loci [13]. The *P. vivax* multidrug resistance 1 gene (*pvmdr1*) and the chloroquine resistance transporter gene (*pvcrt-o*) are orthologous to two genes described in *P. falciparum*, the multidrug resistance 1 gene (*pfmdr1*) and the chloroquine resistance transporter gene (*pfcrt*), which have been identified as potential markers for CQR in *P. vivax* [14].

The *pvcrt-o* gene, described about 20 years ago [15], emerged as a candidate marker of drug resistance and in contrast to *pfcrt*, only a few SNPs (~10) have been described in *pvcrt-o*, and most of them were related but occurring at low frequencies [16]. Although the sequence polymorphism in the *pvcrt-o* locus is relatively limited, a lysine (AAG) insertion in the first exon (amino acid 10), originally discovered in Southeast Asian strains, has been found to be associated with a significant reduction in chloroquine (CQ)’s half-maximal inhibitory concentration (IC50) [13,17,18]. Thenceforth, studies have demonstrated the presence of the K10 insertion in parasites from Southeast Asia and South America [16,19,20].

In the context of *pvmdr1*, this gene was identified in 2005 and, due to its strong sequence similarity to *pfmdr1*, became one of the most important candidate genes investigated in drug susceptibility studies of *P. vivax* [16]. Since then, several SNPs have been identified as potential molecular markers for CQ resistance in *P. vivax*, including T958**M**, Y976**F** and F1076**L**, which are non-synonymous amino acid mutations associated with resistance to CQ [13,14,20,21]. Judging by their protein sequence, the 3 *pvmdr1* mutations are located in 9 to 11 domains of the hydrophobic transmembrane [22]. The presence of these polymorphisms at codons Y976**F** and F1076**L** was registered in malaria-endemic areas where CQ was being used as the first-line antimalarial drug [17,23,24,25]. However, the possibility has been raised that the presence of these two mutations together would be required for the re-emergence of *P. vivax* resistance to CQ [26]. While the Y976F and F1076L polymorphisms are widely distributed in Latin America [27], in Brazil, the increased expression of *pvcrt-o* and *pvmdr1* has been associated with *P. vivax* resistance to CQ [20].

Considering that knowledge of CQ-chemoresistant *P. vivax* circulating parasites in Brazilian endemic areas is crucial for predicting the spread of resistant phenotypes and the need for the introduction of alternative therapies, the aim of this study was to investigate the polymorphisms in the *pvcrt-o* and *pvmdr1* genes to identify their potential predictive role of the CQR phenotype in *P. vivax* samples from Brazilian endemic areas.

## 2. Materials and Methods

### 2.1. Location of the Study and Samples Collected

Samples were collected from January 2018 to August 2022, from *P. vivax*-infected patients in six Amazonian states (Acre, Amazonas, Amapá, Pará, Rondônia and Roraima). Patients were treated and followed clinically and with laboratory testing to verify if they were cured after chemotherapy treatment at the Outpatient Clinic for Acute Febrile Syndromes/Instituto Nacional de Infectologia (INI), which is part of the Reference Center for Malaria Research, Diagnosis and Training—CPD-Mal/Fundação Oswaldo Cruz (FIOCRUZ)/Rio de Janeiro of Extra-Amazonian (22°54′ S 43°12′ W). The treatment adopted for the patients was recommended by the National Program of Malaria Control (PNCM), which comprised the administration of a combination of CQ for 3 days (10 milligrams (mg)/kilogram (kg) on day 1 and 7.5 mg/kg on days 2 and 3) and PQ for 7 days (0.5 mg/kg/day). If patients returned, they were evaluated on days 0, 1, 2, 3, 7, 14, 28 and 42 and in case of symptoms, at any time during the follow-up period.

**Figure 1:**
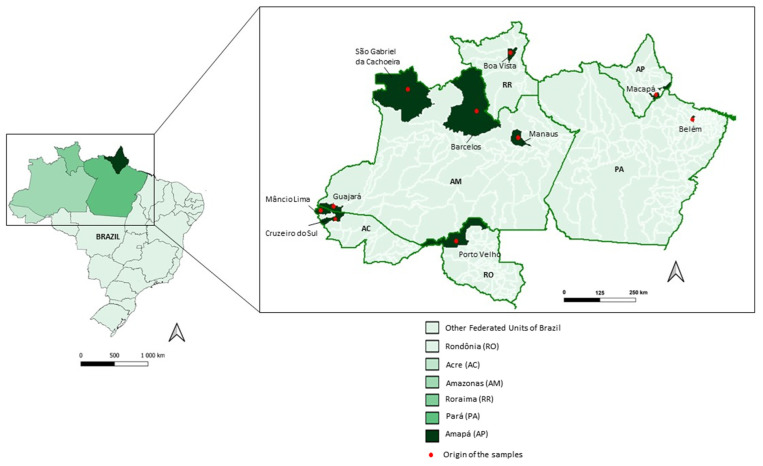
Brazilian map highlighting the Acre (AC), Amazonas (AM), Amapá (AP), Pará (PA), Rondônia (RO) and Roraima (RR) states and the municipalities of parasite infection.

Besides at CPD-Mal, blood samples were also collected in Manaus (3.1190° S, 60.0217° W), the capital of the Amazon state, at the Fundação de Medicina Tropical Doutor Heitor Vieira Dourado (FMT-HVD) and in field conditions in the municipality of Guajará (bordering the Amazonas and Acre states; 02°58’18″ S and 57°40′38′ W), in two municipalities of the Acre state, Cruzeiro do Sul (07°37’50″ S and 72°40’13″ W) and Mâncio Lima (07°36′49″ S and 72°53′47″ W), as well as in the Boa vista municipality (02°49′12″ S and 60°40′23″ W), Roraima state (Table 1 and Figure 1).

### 2.2. Malaria Diagnosis

The malaria diagnoses were made by light microscopy (Giemsa-stained thick blood droplets) in situ. To ensure the presence of a mono-*P. vivax* infection, together with microscopic diagnosis, all samples were subjected to molecular diagnosis by polymerase chain reaction (PCR). Firstly, conventional and real-time PCRs were performed using *Plasmodium* primers [28]. Then, the positive samples were submitted to species-specific single or nested PCRs to detect *P. vivax* [29], *P. falciparum* [30] and/or *P. malariae* [31]. The samples were stored at the Malaria Research Laboratory (LPM) at Instituto Oswaldo Cruz (IOC), headquarters of the Reference Center for Malaria Treatment and Diagnosis (CPD-Mal/Fiocruz). Only patients with *P. vivax* mono-infections were included in the study.

### 2.3. DNA Extraction, Amplification and Sequencing

The DNA from 1 mL blood samples was extracted using the QIAamp™ DNA Blood Midi Kit (QIAGEN, Hilden, Germany), according to the manufacturer’s instructions. For this study, approximately 1186 base pair (bp) fragments of the *pvcrt-o* gene of *P. vivax* were amplified according to the protocol described by Cheong et al. (2020) [14] and a fragment of 800 bp was amplified for the analysis of the SNPs T958**M**, Y976**F** and F1076**L** in the *pvmdr1* gene according to the protocol described by Brega et al. (2005) [32]. PCR products were analyzed by electrophoresis on 2% agarose gel, visualized under a UV transilluminator (DigiDoc-It; UVP, Unpland, CA, USA) and purified using Wizard™ SV Gel and the PCR Clean-Up System (Promega, Madison, WI, USA), following the manufacturer’s procedure. The purified DNA sequencing was carried out through Big Dye™ Terminator Cycle Sequencing Ready Reaction version 3.1 (Applied Biosystems, Carlsbad, CA, USA), with 3.2 μM of forward and reverse PCR primers. DNA sequences to investigate the SNPs in *pvcrt-o* and *pvmdr1* genes were determined using the ABI Prism DNA Analyzer™ 3730 (Applied Biosystems, Carlsbad, CA, USA), at the Fiocruz Genomic Platform PDTIS/Fiocruz RPT01A. Nucleotide sequences were aligned using a ClustalW multiple sequence aligner in BioEdit version 7.7.1 software (North Carolina State University, Raleigh, USA), and the electropherograms were analyzed using NovoSNP^®^ version 3.0.1 software (University of Antwerp, Antwerpen, Belgium), using the quality cutoff set to 10, in order to avoid the lack of real variation, and using the Salvador 1 strain as a reference sequence (GenBank Accession No. AF314649.1 for *pvcrt-o* and GenBank Accession No. AY571984.1 for *pvmdr1*). DNA sequences were deposited in GenBank (the NIH’s genetic sequence database; www.ncbi/nlm/nih.gov/GenBank accessed on 21 August 2023) with the accession numbers OR461289–OR461401.

## 3. Results

### 3.1. Prevalence of Polymorphisms in the pvcrt-o Gene

A total of 104 (80%) of the 130 samples were successfully sequenced for the *pvcrt-o* gene. The insertion of lysine (AAG codon) at position 10, named the K10 insertion and considered a candidate molecular marker of CQR, was detected in 15 (14%) samples: seven from Acre (n = 50; 14%), five from Amazonas (n = 30; 16%), one from Pará (n = 1; 100%) and two from Roraima (n = 19; 11%). Meanwhile, it was not detected in the Rondônia (n = 2) and Amapá (n = 2) (Table 2) samples. In the vast majority of *pvcrt-o* sequenced samples (n = 89; 86%), no mutations were detected and, therefore, these DNA sequences were identical to the Sal-1 strain used as the wild-type reference of CQ-sensitive parasites.

All patients who attended the Malaria Reference Center located in Rio de Janeiro (CPD-Mal) had uncomplicated malaria and were followed up clinically and through laboratory testing; clinical and parasitological cures were confirmed within the expected time frame (Supplementary Materials Table S1). Using the Malaria Epidemiological Surveillance Information System (SIVEP-Malaria data) and Malaria Control Nacional Program (PMCN) definitions, there was no recrudescence in the six patients from the endemic area who had parasites with the K10 insertion until 60 days after initiating treatment of the primary vivax infection. The three patients I, J and L, were considered cases of reinfections and not recrudescence by CQ-resistant parasites due to *P. vivax* notifications >60 days after initiating treatment of the primary vivax infection (Supplementary Materials Table S1).

### 3.2. Prevalence of Polymorphisms in the pvmdr1 Gene

The *pvmdr1* gene was satisfactorily amplified and sequenced in 113 (86%) of the 130 samples investigated. Three-point mutations (T958**M**, Y976**F** and F1076**L**) considered as potentially associated with the *P. vivax* resistance phenotype were examined. The SNP T958**M** was the most prevalent (93%) and was found in parasites from all states. The SNP F1076**L** was present in only three samples from Amazonas (n = 25; 12%), while the Y976**F** mutation was absent in all samples examined (Table 3).

Haplotype analysis of the *pvmdr1* gene showed that almost all samples (n = 104; 92%) had the **M**YF single mutant profile at codon T958**M**, predominating in the states of Acre (58/62; 93%), Amazonas (22/25; 88%) and Roraima (19/21; 90%). In the other states studied, the proportion was less than 2%. The **M**Y**L** double mutant profile (T958**M** + F1076**L**) was detected exclusively in 12% (3/25) of the samples from Amazonas, while the wild-type TYF was detected in the minority of samples: four from Acre (n = 62; 5%) and two from Roraima (n = 21; 10%) (Table 4). In this way, the polymorphism in codon F1076**L** was always associated with polymorphism in codon T958**M**.

The three *P. vivax* patients harboring double mutant **M**Y**L** parasites in *pvmdr1*, whose samples were followed up clinically at the CPD-Mal laboratory, were cured within the expected time (Table 5).

### 3.3. Comparative Alignment between Here Presented pvcrt-o and pvmdr1 Sequences of CQ-Sensitive Parasites with Those Reported by Melo et al. (2014) [20]

**Figure 2:**
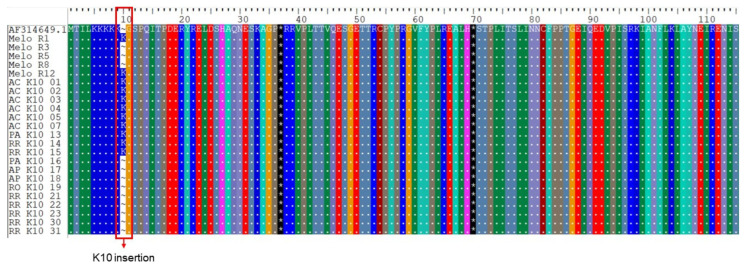
Alignment of representative CQ-resistant and -sensitive *pvcrt-o* sequences, with the K10 insertion highlighted in red. Melo R1 to R12 correspond to CQ-resistant sequences of Melo et al. [20]. The sequences named K10 01 to 31 correspond to the isolates sequenced in this study. AC: Acre; AM: Amazonas; AP: Amapá; PA: Pará; RO: Rondônia; RR: Roraima.

**Figure 3:**
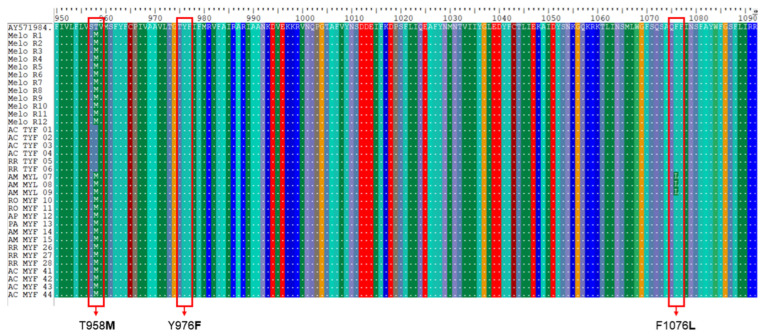
Alignment of representative CQ-resistant and -sensitive *pvmdr1* sequences, with the SNPs investigated highlighted in red (T958**M**, Y976**F** and F1076**L**). Melo R1 to R12 correspond to CQ-resistant sequences of Melo et al. [20]. The sequences named TYF correspond to the isolates with the wild-type haplotype sequenced in this study. Sequences named **M**Y**L** and **M**YF correspond to the isolates with one and two non-synonymous mutations detected in this study, respectively. AC: Acre; AM: Amazonas; AP: Amapá; PA: Pará; RO: Rondônia; RR: Roraima.

When we analyzed our CQ-sensitive sequences with those that were CQ-resistant of Melo et al. [20], we found that 14% of the sensitive *pvcrt-o* gene sequences had the K10 insertion against only two resistant isolates presenting the K10 insertion (Figure 2). In relation to the *pvmdr1* gene, 92% of CQ-sensitive isolates presented the single mutant **M**YF haplotype whereas 100% of CQ-resistant or -sensitive sequences [20] contained the **M**YF haplotype (Figure 3).

## 4. Discussion

*P. vivax* is the most geographically widespread pathogen of human malaria and is responsible for most cases outside the African continent [1]. Vivax malaria is considered a public health problem in many parts of the world, particularly with regard to the morbidity of children and pregnant women [33], the rare but real possibility of fatal infections [16] and the presence of CQ-resistant *P. vivax* parasites [5]. Due to the biological characteristics of *P. vivax* that prevent a continuous in vitro culture system, molecular monitoring has become the tool of choice for the surveillance of resistance to antimalarials due to its practical and economic advantages over in vivo fieldwork and in vitro assays, recommended by the WHO [1,13]. Previous work has shown a significant association between the allele *pfcrt* and *pfmdr1* variants and *P. falciparum* drug resistance [25]. However, putative mutations in these genes, considered as candidate markers of antimalarial drug resistance, are not clear with respect to *P. vivax* [34,35]. In fact, regardless of the chemoresistance phenotype, the genetic polymorphism of *P. vivax* is notable [36] and this parasite is known to be more diverse than *P. falciparum* [37]. As, so far, little is known about the genotype of *P. vivax* parasites circulating in Brazilian endemic areas, thus, it is quite pertinent to investigate the polymorphisms of *pvmdr1* and *pvcrt-o*.

The main gene related to *P. vivax* CQ resistance is *pvcrt-o*. Indeed, the presence of the “AAG” lysine insert in exon I of *pvcrt-o* (referred as the “K10 insert”) has been associated with a significant reduction in the IC50 of CQ [18]. In our analysis, it was possible to identify the K10 insertion in 15 samples (14%) distributed among the states of Acre (7), Amazonas (5), Pará (1) and Roraima (2). This SNP was also reported in studies from French Guiana (57%) [38], China (32%) [39] and, to a lesser extent, India (5.6%) [40], Pakistan (16%) [41] and Thailand (18%) [42]. Due to the scarcity of previous studies, we do not have knowledge about the expansion or retraction of this mutation in Brazil. One possibility would be that this mutation has been introduced to Brazil through the events of illegal mining in French Guiana, where this insertion is frequent, although not directly associated with CQR [38]. In fact, when we analyzed the results of samples from patients who carried *P. vivax* with the K10 insertion, a good response to the CQ treatment occurred within the 28-day expected period, with no observed case of recrudescence. Thus, excluding the small possibility of primaquine acting against minority populations of CQR parasite clones, our data reinforce that the K10 insertion is not a predictor of the *P. vivax* resistance phenotype to treatment with CQ, as previously suggested [13,43,44].

Regarding the *pvmdr1* gene, several studies describe that the substitution in the Y976**F** codon, which changes the amino acid tyrosine to phenylalanine, would be associated with a reduced susceptibility to CQ [39,45,46,47,48]. This SNP has even been identified in endemic areas of China, Cambodia and Ethiopia [19,24,47], where treatment failure has been reported in patients with vivax malaria treated with CQ. Although this SNP was not detected in the present study nor in 2018 in the triple border region involving Colombia, Peru and Brazil/Amazonas [49], previous studies by our group reported the Y976**F** polymorphism at a high frequency (85.7%) in 2009 [26] and at a low frequency (15%) from 2010 to 2014 [50] in the endemic areas of the Legal Amazon. These facts seem to indicate two possibilities of events: either the SNP Y976**F** was present in only a few locations in the endemic region of the Legal Amazon or this mutation was not becoming fixed in the Brazilian endemic region. On the other hand, since we did neither detect the Y976**F** polymorphism in any of the samples here examined, nor any therapeutic failure to CQ in this casuistic, we cannot draw a conclusion about the role of this polymorphism as a marker of *P. vivax* CQR.

In addition to Y976**F**, we also investigated SNPs at the T958**M** and F1076**L** codons in the *pvmdr1* gene, both of which have also been proposed to be potentially associated with CQ resistance [20,34]. The SNP at codon T958**M** was present in almost all analyzed samples (92%), including those of the 34 patients whose parasite genes could be sequenced and responded to CQ treatment within the expected period. This high frequency has been verified by previous studies carried out by our group in Brazilian endemic areas [26,50], as well as by other authors analyzing samples from Africa [51], South America [50] and Southeast Asia [19], indicating the fixation of T958**M** in parasitic populations of *P. vivax* circulating in Brazil and in the world. This fact indicates a low potential of this polymorphism as a CQR marker.

It has been mentioned that the T958**M** and F1076**L** polymorphisms alone may not be associated with CQR, unless if acting together, they could modify the protein’s conformation and enable drug evasion [32,51]. When analyzing these double mutants, we verified that the F1076**L** polymorphism was always present with T958**M**, originating the **M**Y**L** double mutant haplotype. This dependency relationship between these alleles was already noted by our group in isolated parasites from Acre, Amazonas, Rondônia and in autochthonous cases from the Atlantic Forest of Rio de Janeiro [50], as well as by other authors in Southeast Asia [52,53,54], where there are cases of *P. vivax* resistant to CQ treatment. However, once again, the good therapeutic response in patients carrying **M**Y**L** parasites in the present study, disregarding the small possibility of primaquine acting in minority clones of CQR parasites, indicates that the association of double mutants with *P. vivax* CQR is unlikely.

As the Y976F mutant allele was not detected in our series, it was not possible to establish a relationship between chemoresistance and CQ for the TYL double mutant haplotype (T958**M** + Y976**F** + F1076**L**), as previously suggested in the literature [39,55,56].

Finally, the tendency toward tolerance or resistance to the drugs recommended for the treatment of vivax malaria may be more complex, including other genes in addition to *P. falciparum*, where *pvmdr1* SNPs located in the transmembrane domain are associated with CQ vacuole efflux. It is noteworthy that striking differences in the topologies and numbers of SNPs in these transporter genes between *P. vivax* and *P. falciparum* reinforce the idea that mechanisms other than mutations may explain this CQ-resistant phenotype in *P. vivax*. In fact, the *P. vivax* CQ resistance process may also differ by its combined CQ/PQ treatment regimen, so it could be only a matter of time before more CQ-resistant *P. vivax* cases appear.

## 5. Conclusions

The results presented in this study reinforce that the mutations here investigated on *pvcrt-o* and *pvmdr1* may not be good markers of *P. vivax* chemoresistance to CQ. New approaches for the identification of robust genetic markers for monitoring chloroquine resistance in *P. vivax* populations are needed.

## Figures and Tables

**Table 1:** Localities of *P. vivax* parasite blood collection by Brazilian states.

State of Infection	Sample Collection			
	CPD-Mal ^1^	Amazonas	Acre	Roraima
		(FMT-HVD ^2^ and GJ ^3^)	(CZS ^4^ and ML ^5^)	(BV ^6^)
Acre	15	–	52	–
Amazonas	18	12	–	–
Amapá	3	–	–	–
Pará	2	–	–	–
Roraima	6	–	–	20
Rondônia	2	–	–	–

^1^ CPD-Mal: Reference Center for Malaria Treatment and Diagnosis of Brazilian Ministry of Health; ^2^ FMT-HVD: Fundação de Medicina Tropical Doutor Heitor Vieira Dourado, Amazonas state; ^3^ GJ: Guajará municipality, Amazonas state; ^4^ CZS: Cruzeiro do Sul municipality, Acre state; ^5^ ML^: Mâncio Lima municipality, Acre state; ^6^ BV: Boa Vista municipality, Roraima state.

**Table 2:** Distribution of the K10 insertion (AAG codon) in the *pvcrt-o* gene in 104 samples of *P. vivax* from the Legal Amazon.

Gene	SNP	Locality						NT ^1^ (%)
		Acre n = 50	Amazonas n = 30	Amapá n = 2	Pará n = 1	Roraima n = 19	Rondônia n = 2	
*pvcrt-o*	Wild-type	43 (86%)	25 (83%)	2 (100%)	0	17 (89%)	2 (100%)	89 (86%)
	K10 insertion	7 (14%)	5 (16%)	0	1 (100%)	2 (11%)	0	15 (14%)

^1^ NT: total number of samples investigated.

**Table 3:** Frequency of alleles in the *pvmdr1* gene in 113 samples of *P. vivax*, according to the malaria diagnosis localities.

Gene	SNP ^1^	Locality						NT ^2^ (%)
		Acre n = 62	Amazonas n = 25	Amapá n = 2	Pará n = 1	Roraima n = 21	Rondônia n = 2	
*pvmdr1*	Wild-type	4 (5%)	0	0	0	2 (10%)	0	6 (5%)
	958M	58 (93%)	25 (100%)	2 (100%)	1 (100%)	19 (90%)	2 (100%)	107 (95%)
	958M + 1076L	–	3 (12%)	0	0	0	0	3 (3%)

^1^ The bold character represents a non-synonymous mutation detected. ^2^ NT: total number of samples investigated.

**Table 4:** Distribution of haplotypes in the *pvmdr1* gene in 113 samples of *P. vivax* from the Legal Amazon.

Gene	Haplotype ^1^	Mutated Codon	NT ^2^ (%)
*pvmdr1*	**M**YF	1	104 (92%)
	**M**Y**L**	2	3 (3%)
	TYF	0	6 (5%)

^1^ The bold character represents a non-synonymous mutation detected. ^2^ NT: total number of samples investigated.

**Table 5:** Patients followed up at CPD-Mal, carrying *P. vivax* parasites containing the **M**Y**L** double *pvmdr1* mutant haplotype infected in the Amazonas state and the dates of diagnosis and of cure (parasitological and molecular negative assays).

Patient	Date of Diagnosis	Date of Cure ^1^
A	31 December 2021	5 January 2022
B	4 March 2022	11 March 2022
C	14 August 2021	23 August 2021

^1^ Cure: based on parasitological and molecular negative assays.

## Data Availability

Data supporting the conclusions of this article are included within the article. The datasets used and/or analyzed during the present study are available from the corresponding author upon reasonable request.

## Supplementary Materials

The following supporting information can be downloaded at: https://www.mdpi.com/article/10.3390/biomedicines12010141/s1, Table S1: Patients followed up at CPD-Mal and SIVEP-Malaria Database Platform, carrying *P. vivax* parasites containing the insertion of K10 in *pvcrt-o*, according to the state of infection.
