# Supplementary material for: Are pvcrt-o and pvmdr1 Gene Mutations Associated with Plasmodium vivax Chloroquine-Resistant Parasites?
Source: Biomedicines. 2024 Jan 9;12(1):141. doi: 10.3390/biomedicines12010141 (PMC10812985; doi:10.3390/biomedicines12010141)
Supplement: Supplementary file 1 [file biomedicines-12-00141-s001.zip › biomedicines-2738194-supplementary.pdf]

biomedicines

MDPI

Supplementary Material

Table S1. Patients followed- up at CPD-Mal and SIVEP-Malaria database Platform, carrying *P. vivax* parasites containing the insertion of K10 in *pvcrt-o*, according to the state of infection.

	Patients	State of infection	Date of diagnosis	Days until cure[1]
CPD-Mal follow-up	A	Acre	02/26/2018	14
	B	Pará	09/01/2018	19
	C	Roraima	02/17/2020	3
	D	Roraima	08/29/2020	3
	E	Amazonas	09/12/2020	7
	F	Amazonas	11/17/2020	6
	G	Amazonas	01/05/2021	8
	**Patients**	**State of infection**	**Date of first diagnosis notification**	**Days until last diagnosis notification**
SIVEP-Malaria database Platform	H	Acre	07/12/2016	-
	I	Acre	01/20/2018	178
	J	Acre	07/18/2018	121
	K	Acre	07/21/2018	-
	L	Acre	02/15/2018	159
	M	Acre	07/26/2018	-

[1] cure: parasitological and molecular negative assays
